# College student sleep quality and mental and physical health are associated with food insecurity in a multi-campus study

**DOI:** 10.1017/S1368980021001191

**Published:** 2021-09

**Authors:** Rebecca L Hagedorn, Melissa D Olfert, Lillian MacNell, Bailey Houghtaling, Lanae B Hood, Mateja R Savoie Roskos, Jeannine R Goetz, Valerie Kern-Lyons, Linda L Knol, Georgianna R Mann, Monica K Esquivel, Adam Hege, Jennifer Walsh, Keith Pearson, Maureen Berner, Jessica Soldavini, Elizabeth T Anderson-Steeves, Marsha Spence, Christopher Paul, Julia F Waity, Elizabeth D Wall-Bassett, Melanie D Hingle, E Brooke Kelly, J Porter Lillis, Patty Coleman, Mary Catherine Fontenot

**Affiliations:** 1 Davis College of Agriculture, Natural Resources, & Design, West Virginia University, 1194 Evansdale Drive, Agricultural Sciences Building, Room G025, Morgantown, WV26506, USA; 2 Department of Public Health, Campbell University, Buies Creek, NC, USA; 3 School of Nutrition and Food Sciences, Louisiana State University AgCenter, Baton Rouge, LA, USA; 4 Department of Nutrition, Health, & Human Performance, Meredith College, NC, USA; 5 Department of Nutrition, Dietetics & Food Sciences, Utah State University, Logan, UT, USA; 6 Department of Dietetics & Nutrition, The University of Kansas Medical Center, Kansas City, KS, USA; 7 Sauk Valley Community College, Dixon, IL, USA; 8 Department of Human Nutrition & Hospitality Management, The University of Alabama, Tuscaloosa, AL, USA; 9 Department of Nutrition & Hospitality Management, The University of Mississippi, University, MS, USA; 10 Department of Human Nutrition, Food, & Animal Sciences, University of Hawai‘i at Mānoa, Honolulu, HI, USA; 11 Department of Health & Exercise Science, Appalachian State University, Boone, NC, USA; 12 Department of Health Professions, James Madison University, Harrisonburg, VA, USA; 13 Department of Nutrition & Dietetics, School of Public Health, Samford University, Birmingham, AL, USA; 14 School of Government, University of North Carolina at Chapel Hill, Chapel Hill, NC, USA; 15 Center for Health Promotion & Disease Prevention, Gillings School of Global Public Health, University of North Carolina at Chapel Hill, Chapel Hill, NC, USA; 16 Department of Nutrition, University of Tennessee, Knoxville, TN, USA; 17 Department of Public Administration, North Carolina Central University, Durham, NC, USA; 18 Department of Sociology & Criminology, University of North Carolina Wilmington, Wilmington, NC, USA; 19 School of Health Sciences, Nutrition & Dietetics Program, Western Carolina University, Cullowhee, NC, USA; 20 Department of Nutritional Sciences, University of Arizona, Tucson, AZ, USA; 21 Department of Sociology & Criminal Justice, University of North Carolina at Pembroke, Pembroke, NC, USA; 22 Cooperative Research, Extension, & Education Services, Northern Marianas College, Saipan, MP, Northern Mariana Islands; 23 School of Human Ecology, Louisiana Tech University, Ruston, LA, USA

**Keywords:** Food insecurity, Sleep, Mental health, Physical health, College, University, Student

## Abstract

**Objective:**

To assess the relationship between food insecurity, sleep quality, and days with mental and physical health issues among college students.

**Design:**

An online survey was administered. Food insecurity was assessed using the ten-item Adult Food Security Survey Module. Sleep was measured using the nineteen-item Pittsburgh Sleep Quality Index (PSQI). Mental health and physical health were measured using three items from the Healthy Days Core Module. Multivariate logistic regression was conducted to assess the relationship between food insecurity, sleep quality, and days with poor mental and physical health.

**Setting:**

Twenty-two higher education institutions.

**Participants:**

College students (*n* 17 686) enrolled at one of twenty-two participating universities.

**Results:**

Compared with food-secure students, those classified as food insecure (43·4 %) had higher PSQI scores indicating poorer sleep quality (*P* < 0·0001) and reported more days with poor mental (*P* < 0·0001) and physical (*P* < 0·0001) health as well as days when mental and physical health prevented them from completing daily activities (*P* < 0·0001). Food-insecure students had higher adjusted odds of having poor sleep quality (adjusted OR (AOR): 1·13; 95 % CI 1·12, 1·14), days with poor physical health (AOR: 1·01; 95 % CI 1·01, 1·02), days with poor mental health (AOR: 1·03; 95 % CI 1·02, 1·03) and days when poor mental or physical health prevented them from completing daily activities (AOR: 1·03; 95 % CI 1·02, 1·04).

**Conclusions:**

College students report high food insecurity which is associated with poor mental and physical health, and sleep quality. Multi-level policy changes and campus wellness programmes are needed to prevent food insecurity and improve student health-related outcomes.

College is often an experience of new-found autonomy for many students, resulting in lifestyle behaviours changes^(1,2)^ and increases in poor mental health for incoming students^(3)^. Poor mental and physical health can impact college students’ success through poor academic performance and high attrition rates^(4,5,6,7)^. Regarding student well-being, sleep is often impacted upon entering college^(8)^, where between 40 and 65 % of US college students meet the cut-off criteria for poor sleep when measured by the Pittsburgh Sleep Quality Index (PSQI), a validated tool that measures dimensions of sleep quality including duration and continuity^(9)^. This change in sleep quality is often attributed to increased social and academic demands that may result in less or disrupted sleep^(8,10)^, although college students are also at risk for sleep disorders such as Restless Legs Syndrome (RLS).^(11)^ Poor sleep quality is a common symptom of many mental health disorders^(3,12)^, including depression and anxiety^(8,13)^, and can lead to poor physical health as well^(13)^. Further, lack of quality sleep can interfere with college students’ academic achievement^(11)^. Thus, maintaining positive mental and physical well-being with quality sleep in college is essential for student success.

In addition, student well-being can be influenced by experiencing food insecurity, defined as having limited or uncertain access to or availability of safe and nutritious food^(14)^. College food insecurity has also been associated with decreased success in academia^(15)^, making it an area of concern as university administrators seek to improve student retention. Research has emerged in the past decade on the heightened prevalence of food insecurity on college campuses with a recent scoping review reporting a 41 % prevalence rate from peer-reviewed studies, based on weighted means and sample sizes of studies^(16)^. Some evidence suggests that there is a two-way relationship between food security and mental and physical health. Individuals who have poor mental and physical health may be at increased risk for food insecurity due to difficulties with sustained employment and income or lack of financial management^(17)^. At the same time, specifically among college students, being food insecure has been shown to increase the risk of mental health disorders^(18)^ and be associated with self-reported poor health outcomes^(15)^. Further, food insecurity is associated with poor sleep outcomes including longer sleep latency and shorter sleep duration^(12,19)^, although much of this previous research has been limited to the young adult population as a whole.

To our knowledge, researchers in three studies have begun to explore the relationship between food insecurity and sleep among college students, all reporting that food insecurity is related to poor sleep among college students, although the methods used to measure food insecurity and sleep quality vary in these studies as well as the sample size^(20,21,22)^. El Zein and colleagues (2019) used validated tools and captured data from multiple institutions; however, this research only targeted first-year students^(21)^, thus excluding student populations that are typically more at risk for food insecurity^(15)^. The objective of this study was to explore the association between food insecurity, perceived mental and physical well-being, and sleep quality among college students across twenty-two colleges and universities. We hypothesised that food insecurity would be associated with a greater risk of poor sleep quality and increased days with mental and physical health issues.

## Methods

### Participants and procedures

In the fall of 2019, a cross-sectional, online survey was distributed to college students across twenty-two colleges and universities in the US and territories. The twenty-two colleges and universities that participated were part of a previous food insecurity working group^(15)^, with additional partners recruited at the 2019 Southeastern University Consortium On Hunger, Poverty And Nutrition conference and through peer networks. All colleges and universities that had a principal investigator interested were able to participate. The twenty-two institutions were located in twelve states: Alabama, Arizona, Hawaii, Illinois, Kansas, Louisiana, Mississippi, North Carolina, Tennessee, Utah, Virginia and West Virginia.

Institutional Review Board approval was granted at the lead university (approval no. 1904527720A003) and covered all participating institutions. College students had to be enrolled at a participating university or college at the time of the study and at least 18 years old to participate. There was no cut-off for age. Online consent was required before college students could access the online Qualtrics survey (Qualtrics). College students were recruited via email through campus listservs direct to students or email to departmental contacts on campus to forward to students. Two universities offered no incentive for participation, but the remaining institutions offered either a drawing or random selection for a limited number of gift cards or course extra credit. The recruitment email included a link to the online Qualtrics survey and took college students an estimated 30 min to complete. The survey contained 122 questions and was developed by the authors through modification of previously used food insecurity surveys^(15,23,24)^ and guided by college food insecurity literature, including the Government Accountability Office report on college hunger^(25)^. Only variables related to sleep and mental and physical health were included in this analysis.

## Measures

### Food security status

Food-insecure students were identified using the United States Department of Agriculture Adult Food Security Survey^(26)^, as used in previous college food insecurity literature^(15,23,27)^. This validated ten-item survey tool asked students to respond affirmatively or non-affirmatively to statements measuring several characteristics of food insecurity, including the inability to acquire food for financial reasons, concern over obtaining food, and reduced quality and quantity of food consumed. The phrasing was modified from the original United States Department of Agriculture tool which assesses food insecurity ‘in the last 12 months’ to ‘since being in college’ to ensure questions were focused on campus issues instead of when students may have lived at home with a parent or guardian as a minor^(28)^. Using the United States Department of Agriculture protocol^(29)^, students were categorised into high (0 affirmative responses), marginal (1–2 affirmative responses), low (3–5 affirmative responses) and very low food security (6–10 affirmative responses) categories. Students were further dichotomised as food secure (high and marginal food security) and food insecure (low and very low food security).

### Sleep quality

The PSQI, a nineteen-item, validated questionnaire, was used to measure sleep quality over the past month^(30,31)^ and has been implemented previously among college students^(13,21,32)^. The first four questions asked students to report the time they went to bed (not necessarily the time they fell asleep), the number of minutes it took to fall asleep, when they awoke and hours of sleep per night. The next ten questions asked how often the participant had trouble sleeping because of reasons such as having to get up to use the restroom, feeling too hot or too cold, having pain, or waking up in the middle of the night, with questions answered on a four-point scale ranging from ‘never’ to ‘three times or more a week.’ Students also rated on the same four-point scale their use of medication to fall asleep, how often they have trouble staying awake during social activity and if enthusiasm has been lacking to complete tasks. Last, students provided a subjective rating of their sleep quality on a four-point scale from ‘very good’ to ‘very bad.’ The PSQI questions were combined into seven different scores ranging from 0 (no difficulty) to 3 (severe difficulty) on topics of sleep quality, sleep latency, sleep duration, sleep efficiency, sleep disturbances, sleep medication and daytime dysfunction per the PSQI scoring guidelines^(30)^. The seven component scores were summed for a final PSQI score that ranged from 0 to 21, with sleep quality declining with each increase in the score^(30)^. PSQI scores >5 are indicative of poor sleep quality^(30)^.

### Physical and mental health

Three items from the Centers for Disease Control and Prevention’s four-item Healthy Days Core Module, a component of the Centers for Disease Control and Prevention’s Health-Related Quality of Life measure, were used^(33,34)^. The three items from the Healthy Days Core Module asked the students about the number of days in the past 30 d they have experienced particular health-related conditions or problems. The first and second questions assessed the number of impaired physical or mental health days in the past month, and the third assessed limitations in usual activities due to poor physical or mental health. All questions were scored between 0 and 30 d with higher days indicating more occurrence of poor mental or physical health.

### Covariates

University attended, age, sex and race of participants were self-reported. Height and weight were self-reported and used to calculate BMI. Information on whether a student lived on or off campus, had dependents and had a disability were collected due to their relationships with food insecurity^(25)^. Last, students completed questions regarding RLS due to the relationship with sleep quality^(11)^ and mental and physical health^(35,36)^. The 2003 criteria for the diagnosis of RLS from the International Restless Legs Syndrome Study Group were used^(37)^. Per these criteria, students responded to five questions to assess four criteria of RLS^(38)^. The first criterion composed of two questions asking: ‘Do you have, or have you had, recurrent uncomfortable feelings or sensations in your legs while you are sitting or lying down?’ and ‘Do you, or have you had, a recurrent need or urge to move your legs while you are sitting or lying down?’ A ‘yes’ response to both questions was required for RLS affirmation in the first criterion. The second criterion asked, ‘Are you more likely to have these feelings when you are resting (either sitting or lying down) or when you are physically active?’ with a ‘resting’ response required for RLS affirmation. The third criterion asked, ‘If you get up or move around when you have these feelings, do these feelings get any better while you actually keep moving?’ to which a participant responded ‘yes’ for RLS affirmation. Last, respondents who answered ‘evening’ and/or ‘night’ to the question ‘At which times of day are these feelings in your legs most likely to occur?’ were considered to meet the fourth criterion. Participants were considered to have symptoms of RLS if they responded affirmatively to all four criteria, creating a dichotomised variable of positive or negative for RLS symptoms.

### Analysis

Sample size at each university is shown in Table 1. Only respondents that completed the full Adult Food Security Survey, PSQI and Healthy Days Core Module questions were included in the study and combined across universities for the analytical sample (*n* 17 686). Categorical variables are presented as numbers and percentages; these were compared by food security status using the Pearson’s *χ* ^2^ test and Cramer’s V for effect size. Continuous variables are presented as mean and standard deviation; these were compared by food security status using the Wilcoxon test and Pearson’s *r* for effect size. The PSQI and physical and mental health variables were entered into a logistic regression model to identify independent factors for food insecurity. A second model was conducted with the covariates university, age, sex, race, BMI, living on or off campus, having dependents, having a disability and having RLS symptoms added to calculate adjusted OR (AOR) (*n* 16 793). A sensitivity analysis was conducted using a third model that included only freshman level students and controlled for the aforementioned covariates from the second model (*n* 3098). Goodness of fit was assessed and showed the likelihood ratio tests for all models at *P* < 0·0001, indicating good model fit for all models. Statistical significance was deemed at *P* < 0·004 using Bonferroni correction. Statistical analysis was performed using JMP Pro version 12.2 (SAS Institute Inc.) and SAS version 9.4 software (SAS Institute Inc.).

**Table 1:** Sample from participating universities

University	*n*
University 1	2655
University 2	3268
University 3	2577
University 4	257
University 5	373
University 6	343
University 7	2248
University 8	118
University 9	660
University 10	136
University 11	519
University 12	211
University 13	318
University 14	1290
University 15	135
University 16	3148
University 17	373
University 18	73
University 19	484
University 20	144
University 21	2687
University 22	134

## Results

The analytical sample (*n* 17 686) is described in Table 2. Students were predominantly white (83·5 %) and female (74·8 %), with an average 8·1 (9·0)/30 d of poor mental health, 3·2 (5·7)/30 d of poor physical health and 3·9 (6·3)/30 d when their physical or mental health interfered with completing daily tasks. Mean age was 22·4 (5·5) years and mean BMI was 24·7 (5·5). Most students lived off campus (66·9 %), had no dependents (92·4 %), no disabilities (92·0 %) and no RLS symptoms (94·1 %). Students’ mean PSQI score was 7·2 (3·5) indicating poor sleep quality. Many (43·4 %) college students were food insecure. In univariate analysis, food insecurity status was associated with being Black or mixed race (*χ* ^2^ = 215·17, *P* < 0·0001), living off campus (*χ* ^2^ = 159·87, *P* < 0·0001), having a self-reported disability (*χ* ^2^ = 178·26, *P* < 0·0001) and having RLS symptoms (*χ* ^2^ = 63·56, *P* < 0·0001). Students who were food insecure had higher PSQI scores (*t* = 40·20, *P* < 0·0001) and a higher number of days with poor mental health (*t* = 37·62, *P* < 0·0001), physical health (*t* = 19·72, *P* < 0·0001) and days in which their mental and physical health interfered with their daily activities (*t* = 35·33, *P* < 0·0001).

**Table 2:** Characteristics of college student participants and correlation with food security status

Variable	Full sample *n*	%	Food secure *n*	%	Food insecure *n*	%	*P*	Effect size
Food security status								
Food secure	10 013	56·6						
Food insecure	7673	43·4	–	–	–	–	–	–
Sex								
Male	4447	25·2	2574	25·7	1873	24·4		
Female	13 220	74·8	7428	74·3	5792	75·6	0·1157	0·0106
Race								
White	14 689	83·5	8507	85·4	6182	81·1		
Black	869	5·0	379	3·8	490	6·4		
Asian	895	5·1	566	5·7	329	4·3		
Biracial or other	1129	6·4	510	5·1	619	8·1	<0·0001	0·1023
Living								
On campus	5860	33·1	3674	36·7	2186	28·5		
Off campus	11 822	66·9	6338	63·3	5484	71·5	<0·0001	0·0850
Has dependents								
No	16 331	92·4	9264	92·6	7067	92·2		
Yes	1344	7·6	742	7·4	602	7·8	0·1762	0·0091
Has a self-reported disability							<0·0001	0·0897
No	16 260	92·0	9424	94·2		89·1		
Yes	1418	8·0	584	5·8		10·9		
Restless leg syndrome symptoms							<0·0001	0·0579
No	16 566	94·1	9510	95·2	7056	92·5		
Yes	1048	5·9	474	4·8	574	7·5		

	Full sample		Food secure		Food insecure			
	Mean	sd	Mean	sd	Mean	sd	*P*	
Age (years)	22·4	5·5	22·5	5·9	22·3	5·0	<0·0001	−0·02243
BMI (kg/m^2^)	24·7	5·5	24·4	5·0	25·2	5·9	<0·0001	0·07245
PSQI	7·2	3·5	6·3	3·2	8·3	3·6	<0·0001	0·28670
HDCM physical health (d)	3·2	5·7	2·5	5·0	4·2	6·4	<0·0001	0·14030
HDCM mental health (d)	8·1	9·0	6·1	7·8	10·9	9·8	<0·0001	0·26071
HDCM health limitations (d)	3·9	6·3	2·6	4·9	5·7	7·4	<0·0001	0·24559

PSQI, Pittsburgh sleep quality index; HDCM, Healthy Days Core Module.

OR and AOR, controlling for covariates, from the logistic regression models are shown in Table 3. When controlling for covariates, being food insecure was associated with higher PSQI scores (AOR: 1·13; 95 % CI 1·12, 1·14), and increased days with poor physical health (AOR: 1·01; 95 % CI 1·01, 1·02) and mental health (AOR: 1·03; 95 % CI 1·02, 1·03). Experiencing food insecurity was also associated with more days where students’ mental or physical health limited their ability to complete daily tasks (AOR: 1·03; 95 % CI 1·02, 1·04). The sensitivity analysis model containing only freshman is also shown in Table 3.

**Table 3:** Food insecurity odds in relationship with sleep quality, physical health and mental health (*n* 17 686)*,†

Variable	Food insecurity		Food insecurity		Freshman only food insecurity	
	OR	95 % CI	AOR	95 % CI	AOR	95 % CI
PSQI	1·13	1·12, 1·15	1·13	1·12, 1·14	1·12	1·09, 1·15
HDCM physical health	1·01	1·01, 1·02	1·01	1·01, 1·02	1·02	1·01, 1·03
HDCM mental health	1·03	1·02, 1·03	1·03	1·02, 1·03	1·02	1·01, 1·03
HDCM health limitations	1·04	1·03, 1·04	1·03	1·02, 1·04	1·04	1·02, 1·06

AOR, adjusted OR; PSQI, Pittsburgh sleep quality index; HDCM, Healthy Days Core Module.

* AOR model controls for university, age, sex, race, BMI, living on or off campus, having dependents, having a disability and having restless leg syndrome symptoms (*n* 16 560).

† Freshman only AOR model includes only freshman level students and controls for controls for university, age, sex, race, BMI, living on or off campus, having dependents, having a disability and having restless leg syndrome symptoms (*n* 3098).

## Discussion

This study aimed to explore the relationship between food insecurity, sleep quality, and mental and physical health among college students using a multi-campus approach. As hypothesised, food insecurity among college students was associated with worse sleep quality, while food security among the respondents was associated with higher sleep quality. Food insecurity was also associated with an increased number of days with poor mental and physical health and has these health consequences impact completing tasks. These results highlight the impact food insecurity may have on the daily functioning of students, influencing their health and well-being outcomes.

Sleep quality among college students is reportedly low^(9)^. The student population used within this study aligns with these previous findings as the analytical sample has an average PSQI score of 7·2, indicating the population as a whole had poor sleep quality^(30)^. The reasoning for poor sleep quality among college students was explored in a 2010 study by Lund and colleagues that reported emotional and physical stress were predictors of poor quality sleep^(13)^. However, food insecurity was not included as a potential explanation. Our study finds that food-insecure students reported a higher prevalence of RLS, a common deterrent of adequate sleep quality, and overall poorer sleep as indicated by higher PSQI scores among food-insecure students. Controlling for RLS, food insecurity was associated with worsened PSQI scores, indicating that food insecurity may be an important factor influencing students’ overall sleep quality. Becerra and colleagues report that components of food insecurity are a social determinant of sleep health specifically influencing daytime sleepiness and breathing issues during sleep^(22)^. This relationship between food insecurity and poor sleep health was attributed to potential disruptions in glycaemic control resulting in low energy, circadian rhythm disruption and weight issues causing snoring or sleep apnoea^(22)^. More research is needed to understand the mechanisms between food insecurity and overall poor sleep quality among college students.

In a similar study, El Zein *et al.* reported that food-insecure students were 132 % more likely to have poor sleep quality^(21)^. These odds are higher than that reported in this current study, which may have been attributed to the first-year student population assessed. However, sensitivity analysis showed little difference between freshman and the full study population. Therefore, these results may be explained by the Time Preference Theory as explored by Knol and colleges (2017) when seeking to understand food insecurity and poor health outcomes^(39)^. Using this theory, college students with higher time preference, referring to an individual who selects short-term rewards instead of long-term rewards that can impact future health outcomes, may be neglecting their sleep habits to reap the benefit of good grades without consideration of the long-term impact^(40)^. As poor sleep can lead to academic consequences among college students^(41,42)^, higher education administration must promote healthy sleep programming on campus. Further, poor sleep quality can influence the development of chronic disease that can impact college students throughout their lifespan^(43,44)^. Specifically, Martinez and colleagues found that food insecurity reported fewer days with enough sleep which was related to higher weight and poor health outcomes among college students^(20)^, making it imperative to improve sleep quality where possible. Previous research has shown that campus-based sleep campaigns may help students improve their sleep quality^(45)^, although, without the alleviation of food insecurity, these programmes might lack effectiveness.

This study also demonstrates a small but statistically significant correlation between the occurrence of poor mental and physical health days and food insecurity among college students. Previously, college students who were classified as food insecure have been classified as more likely to report their health as fair or poor^(15)^. Mental health outcomes, including depression and anxiety, have also been previously associated with college food insecurity. For example, Wattick, Hagedorn and Olfert found that college students suffered from mental health issues for roughly a third of the month, and the likelihood of having depressive or anxious symptoms was increased among students who were food insecure^(46)^. There is likely a two-way relationship between food insecurity and poorer mental and physical health. Food insecurity may contribute to poor mental health due to increased stress experienced by food-insecure individuals^(47)^ or stigmatisation they face^(48)^. The poorer dietary quality shown among food-insecure populations could also contribute to nutritional deficits that serve as a barrier to healthy functioning, both mental and physical^(46)^. Poor mental and physical health can also contribute to sleep disturbances and lowered productivity^(49)^. Thus, food insecurity, mental and physical health, and sleep quality may be a bidirectional cyclic process. In turn, declines in daily functioning overall could lead to an increased economic burden on food-insecure individuals^(50,51)^.

As food-insecure populations often face financial limitations^(52)^, the additional financial hardship caused by days with poor mental and physical health may inhibit food-insecure individuals from overcoming the barriers they face. Thus, due to the potential bidirectional relationship between food insecurity and poor mental and physical health, a holistic approach is needed on college campuses to promote the health of the mind and body. Further, in light of the coronavirus disease 2019 (COVID-19) pandemic, special attention may be needed for students who face financial burdens as a result of the pandemic. Reports on food insecurity highlight an increased prevalence since the pandemic^(53)^, and college students are reported to have increased poor mental health^(54)^ with decreased access to assistance during the pandemic^(55)^; thus, higher education administrators should continue to provide and advocate for outreach and support to students in need^(56)^.

This study used the largest sample of college students to date to further strengthen the knowledge of the association between food insecurity, mental and physical health, and sleep. College students from twenty-two higher education institutions were represented, thus highlighting students from diverse universities and regions. However, this study is not without limitations. First, the effect size of the OR is small although significant. The non-probability sample may contain a degree of selection bias, and the cross-sectional study design limits the ability to distinguish causation. Self-response bias may have occurred due to the self-reporting of measures. Further, the sample is predominately white and female; thus, future research needs to target male and minority populations to expand the understanding of this issue in all college populations. The validity of the United States Department of Agriculture food security tools when used with college students has been questioned^(57)^, as well as the modality of measurement^(58)^, and thus may cause error in the food insecurity measurement. However, the ten-item Adult Food Security Survey used in this study has been reported to have the best fit among college students^(59)^. Further, the PSQI and the Healthy Days Core Module were measured on a time frame looking at the past 30 d, whereas the Adult Food Security Survey assessed since students began college which may impact results as food insecurity can be episodic in nature. Therefore, future research should seek to assess these variables using the same time frame to ensure students are reporting occurrence of each variable simultaneously.

Food insecurity is a current public health problem facing college students in the US, with this study reporting a 43·4 % prevalence rate among college students at twenty-two higher education institutions. These food-insecure students report declined sleep quality and mental and physical health which could impact their success in college as well as their overall health in the long term. Thus, college and university wellness programmes should aim to support food-insecure students as a means of improving the mental and physical well-being of students and inform the need for holistic programming that considers multiple aspects of college student well-being and health. Further, these results can be used by advocates for college food security to show policymakers the layers of impact food insecurity may have on college students’ welfare.

## References

[1] Owens H, Christian B & Polivka B (2017) Sleep behaviors in traditional-age college students: a state of the science review with implications for practice. J Am Assoc Nurse Pract 29, 695–703. 28981991 10.1002/2327-6924.12520

[2] Pancer SM, Hunsberger B, Pratt MW et al. (2000) Cognitive complexity of expectations and adjustment to university in the first year. J Adolesc Res 15, 38–57.

[3] Ghrouz AK, Noohu MM, Manzar MD et al. (2019) Physical activity and sleep quality in relation to mental health among college students. Sleep Breath 23, 627–634. 30685851 10.1007/s11325-019-01780-z

[4] Taras H (2005) Physical activity and student performance at school. J School Health 75, 214–218. 16014127 10.1111/j.1746-1561.2005.00026.x

[5] Calicchia JA & Graham LB (2006) Assessing the relationship between spirituality, life stressors, and social resources: buffers of stress in graduate students. N Am J Psychol 8, 307–320.

[6] Beauchemin J, Gibbs T & Granello P (2018) Wellness promotion courses in university settings: a review of the outcome research. BHAC J 2, 36–49.

[7] Bruffaerts R, Mortier P, Kiekens G et al. (2018) Mental health problems in college freshmen: prevalence and academic functioning. J Affect Disord 225, 97–103. 28802728 10.1016/j.jad.2017.07.044 PMC5846318

[8] Pilcher JJ, Ginter DR & Sadowsky B (1997) Sleep quality versus sleep quantity: relationships between sleep and measures of health, well-being and sleepiness in college students. J Psychosom Res 42, 583–596. 9226606 10.1016/s0022-3999(97)00004-4

[9] Becker SP, Jarrett MA, Luebbe AM et al. (2018) Sleep in a large, multi-university sample of college students: sleep problem prevalence, sex differences, and mental health correlates. Sleep Health 4, 174–181. 29555131 10.1016/j.sleh.2018.01.001 PMC5863586

[10] Famodu OA, Barr ML, Holásková I et al. (2018) Shortening of the Pittsburgh sleep quality index survey using factor analysis. Sleep Disord 2018, 9643937. 29850262 10.1155/2018/9643937 PMC5925150

[11] Gaultney JF (2010) The prevalence of sleep disorders in college students: impact on academic performance. J Am Coll Health 59, 91–97. 20864434 10.1080/07448481.2010.483708

[12] Nagata JM, Palar K, Gooding HC et al. (2019) Food insecurity is associated with poorer mental health and sleep outcomes in young adults. J Adolesc Health 65, 805–811. 31587956 10.1016/j.jadohealth.2019.08.010 PMC6874757

[13] Lund HG, Reider BD, Whiting AB et al. (2010) Sleep patterns and predictors of disturbed sleep in a large population of college students. J Adolesc Health 46, 124–132. 20113918 10.1016/j.jadohealth.2009.06.016

[14] United States Department of Agriculture Economic Research Service Definitions of food security (2020). https://www.ers.usda.gov/topics/food-nutrition-assistance/food-security-in-the-us/definitions-of-food-security.aspx (accessed April 2020).

[15] Hagedorn RL, McArthur LH, Hood LB et al. (2019) Expenditure, coping, and academic behaviors among food-insecure college students at 10 higher education institutes in the Appalachian and Southeastern Regions. Curr Dev Nutr 3, nzz058. 31149651 10.1093/cdn/nzz058 PMC6536735

[16] Nikolaus CJ, An R, Ellison B et al. (2020) Food insecurity among college students in the United States: a scoping review. Adv Nutr 11, 327–348. 31644787 10.1093/advances/nmz111 PMC7442331

[17] Lent MD, Petrovic LE, Swanson JA et al. (2009) Maternal mental health and the persistence of food insecurity in poor rural families. J Health Care Poor Underserved 20, 645–661. 19648695 10.1353/hpu.0.0182

[18] Bruening M, Brennhofer S, van Woerden I et al. (2016) Factors related to the high rates of food insecurity among diverse, urban college freshmen. J Acad Nutr Diet 116, 1450–1457. 27212147 10.1016/j.jand.2016.04.004 PMC5520984

[19] Ding M, Keiley MK, Garza KB et al. (2015) Food insecurity is associated with poor sleep outcomes among US adults. J Nutr 145, 615–621. 25733479 10.3945/jn.114.199919

[20] Martinez SM, Grandner MA, Nazmi A et al. (2019) Pathways from food insecurity to health outcomes among California university students. Nutrients 11, 1419. 10.3390/nu11061419 PMC6627945 31238534

[21] El Zein A, Shelnutt KP, Colby S et al. (2019) Prevalence and correlates of food insecurity among US college students: a multi-institutional study. BMC Public Health 19, 660. 31142305 10.1186/s12889-019-6943-6 PMC6542079

[22] Becerra MB, Bol BS, Granados R et al. (2020) Sleepless in school: the role of social determinants of sleep health among college students. J Am Coll Health 68, 185–191. 30489219 10.1080/07448481.2018.1538148

[23] McArthur LH, Ball L, Danek AC et al. (2018) A high prevalence of food insecurity among university students in appalachia reflects a need for educational interventions and policy advocacy. J Nutr Educ Behav 50, 564–572. 29242138 10.1016/j.jneb.2017.10.011

[24] Hagedorn RL & Olfert MD (2018) Food insecurity and behavioral characteristics for academic success in young adults attending an Appalachian university. Nutrients 10, 361. 10.3390/nu10030361 PMC5872779 29547533

[25] USGA (2018) Food Insecurity Better Information Could Help Eligible College Students Access Federal Food Assistance Benefits. Washington, DC: USGA.

[26] US Department of Agriculture Economic Research Service U.S. Adult Food Security Survey Module: Three-stage design, with screeners (2012) US Department of Agriculture Economic Research Service. https://www.ers.usda.gov/media/8279/ad2012.pdf (accessed June 2020).

[27] Soldavini J, Berner M & Da Silva J (2019) Rates of and characteristics associated with food insecurity differ among undergraduate and graduate students at a large public university in the Southeast United States. Prev Med Rep 14, 100836. 30886818 10.1016/j.pmedr.2019.100836 PMC6403080

[28] McArthur LH, Fasczewski KS, Wartinger E et al. (2018) Freshmen at a university in Appalachia experience a higher rate of campus than family food insecurity. J Community Health 43, 969–976. 29633107 10.1007/s10900-018-0513-1

[29] Bickel G, Nord M, Price C et al. (2000) Guide to Measuring Household Food Security: Revised 2000. Alexandria, VA: U.S. Department of Agriculture, Food and Nutrition Service.

[30] Buysse DJ, Reynolds CF, Monk TH et al. (1989) The Pittsburgh sleep quality index: a new instrument for psychiatric practice and research. Psychiatr Res 28, 193–213. 10.1016/0165-1781(89)90047-4 2748771

[31] Dietch JR, Taylor DJ, Sethi K et al. (2016) Psychometric evaluation of the PSQI in US college students. J Clin Sleep Med 12, 1121–1129. 27166299 10.5664/jcsm.6050 PMC4957190

[32] Vargas PA, Flores M & Robles E (2014) Sleep quality and body mass index in college students: the role of sleep disturbances. J Am Coll Health 62, 534–541. 24933244 10.1080/07448481.2014.933344 PMC4221412

[33] Hennessy CH, Moriarty DG, Zack MM et al. (1994) Measuring health-related quality of life for public health surveillance. Public Health Rep 109, 665. 7938388 PMC1403555

[34] Health-Related Quality of Life (HRQOL) (2018) Centers for Disease Control and Prevention. Atlanta, GA. https://www.cdc.gov/hrqol/hrqol14_measure.htm (accessed June 2020).

[35] Zhuang S, Na M, Winkelman JW et al. (2019) Association of restless legs syndrome with risk of suicide and self-harm. JAMA Network Open 2, e199966–e199966. 31441941 10.1001/jamanetworkopen.2019.9966 PMC6714009

[36] Kushida C, Martin M, Nikam P et al. (2007) Burden of restless legs syndrome on health-related quality of life. Qual Life Res 16, 617. 17268935 10.1007/s11136-006-9142-8

[37] Allen RP, Picchietti D, Hening WA et al. (2003) Restless legs syndrome: diagnostic criteria, special considerations, and epidemiology: a report from the restless legs syndrome diagnosis and epidemiology workshop at the National Institutes of Health. Sleep Med 4, 101–119. 14592341 10.1016/s1389-9457(03)00010-8

[38] Sunwoo J-S, Kim W-J, Chu MK et al. (2019) Association between restless legs syndrome symptoms and self-reported hypertension: a nationwide questionnaire study in Korea. J Korean Med Sci 34, e130. 31020817 10.3346/jkms.2019.34.e130 PMC6484175

[39] Knol LL, Robb CA, McKinley EM et al. (2017) Food insecurity, self-rated health, and obesity among college students. Am J Health Educ 48, 248–255.

[40] Brown H & Biosca O (2016) Exploring the relationship between time preference, body fatness, and educational attainment. Soc Sci Med 158, 75–85. 27111437 10.1016/j.socscimed.2016.04.016

[41] Hershner SD & Chervin RD (2014) Causes and consequences of sleepiness among college students. Nature Sci Sleep 6, 73. 25018659 10.2147/NSS.S62907 PMC4075951

[42] Trockel MT, Barnes MD & Egget DL (2000) Health-related variables and academic performance among first-year college students: implications for sleep and other behaviors. J Am Coll Health 49, 125–131. 11125640 10.1080/07448480009596294

[43] Altman NG, Izci-Balserak B, Schopfer E et al. (2012) Sleep duration versus sleep insufficiency as predictors of cardiometabolic health outcomes. Sleep Med 13, 1261–1270. 23141932 10.1016/j.sleep.2012.08.005 PMC3527631

[44] Gray L, Lee I-M, Sesso HD et al. (2011) Blood pressure in early adulthood, hypertension in middle age, and future cardiovascular disease mortality: HAHS (Harvard Alumni Health Study). J Am Coll Cardiol 58, 2396–2403. 22115646 10.1016/j.jacc.2011.07.045 PMC3253414

[45] Orzech KM, Salafsky DB & Hamilton LA (2011) The state of sleep among college students at a large public university. J Am Coll Health 59, 612–619. 21823956 10.1080/07448481.2010.520051

[46] Wattick R, Hagedorn R & Olfert M (2018) Relationship between diet and mental health in a young adult Appalachian college population. Nutrients 10, 957. 10.3390/nu10080957 PMC6115820 30044399

[47] Martin M, Maddocks E, Chen Y et al. (2016) Food insecurity and mental illness: disproportionate impacts in the context of perceived stress and social isolation. Public Health 132, 86–91. 26795678 10.1016/j.puhe.2015.11.014

[48] Palar K, Frongillo EA, Escobar J et al. (2018) Food insecurity, internalized stigma, and depressive symptoms among women living with HIV in the United States. AIDS Behav 22, 3869–3878. 29948333 10.1007/s10461-018-2164-8 PMC6209540

[49] Rosekind MR, Gregory KB, Mallis MM et al. (2010) The cost of poor sleep: workplace productivity loss and associated costs. J Occup Environ Med 52, 91–98. 20042880 10.1097/JOM.0b013e3181c78c30

[50] Greenberg PE, Fournier A-A, Sisitsky T et al. (2015) The economic burden of adults with major depressive disorder in the United States (2005 and 2010). J Clin Psychiatr 76, 155–162. 10.4088/JCP.14m09298 25742202

[51] Druss BG, Marcus SC, Olfson M et al. (2001) Comparing the national economic burden of five chronic conditions. Health Aff 20, 233–241. 10.1377/hlthaff.20.6.233 11816664

[52] Gaines A, Robb CA, Knol LL et al. (2014) Examining the role of financial factors, resources and skills in predicting food security status among college students. Int J Consum Stud 38, 374–384.

[53] Niles MT, Bertmann F, Belarmino EH et al. (2020) The early food insecurity impacts of COVID-19. Nutrients 12, 2096. 10.3390/nu12072096 PMC7400862 32679788

[54] Cao W, Fang Z, Hou G et al. (2020) The psychological impact of the COVID-19 epidemic on college students in China. Psychiatr Res 287, 112934. 10.1016/j.psychres.2020.112934 PMC7102633 32229390

[55] Lee J (2020) Mental health effects of school closures during COVID-19. Lancet Child Adolesc Health 4, 421. 32302537 10.1016/S2352-4642(20)30109-7 PMC7156240

[56] Zhai Y & Du X (2020) Addressing collegiate mental health amid COVID-19 pandemic. Psychiatr Res 288, 113003. 10.1016/j.psychres.2020.113003 PMC7162776 32315885

[57] Nikolaus CJ, Ellison B & Nickols-Richardson SM (2019) College students’ interpretations of food security questions: results from cognitive interviews. BMC Public Health 19, 1282. 31604466 10.1186/s12889-019-7629-9 PMC6788030

[58] Nikolaus CJ, Ellison B & Nickols-Richardson SM (2020) Food insecurity among college students differs by questionnaire modality: an exploratory study. Am J Health Behav 44, 82–89. 31783935 10.5993/AJHB.44.1.9

[59] Nikolaus CJ, Ellison B & Nickols-Richardson SM (2019) Are estimates of food insecurity among college students accurate? Comparison of assessment protocols. PLoS One 14, e0215161. 31017912 10.1371/journal.pone.0215161 PMC6481800

